# Supplementary material for: An LLM-assisted framework for accelerated and verifiable clinical hypothesis testing from electronic health records
Source: medRxiv. 2026 Feb 12:2026.02.10.26346008. Preprint. [Version 1] doi: 10.64898/2026.02.10.26346008 (PMC12919150; doi:10.64898/2026.02.10.26346008)
Supplement: 1 [file NIHPP2026.02.10.26346008v1-supplement-1.pdf]

medRxiv preprint doi: https://doi.org/10.64898/2026.02.10.26346008; this version posted February 12, 2026. The copyright holder for this preprint (which was not certified by peer review) is the author/funder, who has granted medRxiv a license to display the preprint in perpetuity.

It is made available under a CC-BY 4.0 International license .

Planner

Objective: Convert the user's free-text research question into a fully structured study design with necessary details.

```
Your goal is to convert the user's free-text request into a structured study design specification.

General rules:
- Organize the content into these components: analysis type, dataset, period of interest, inclusion criteria, exclusion criteria, variables
(note: variable requirements depend on the analysis type).
- If any component is missing, set its value to None.
- Provide complete derivation, categorization, data processing, and calculation details; do not omit steps.
- If a unit appears with a variable name, preserve it exactly as written.
- Preserve all keyword and variable names exactly as provided (including spelling, casing, punctuation, and spacing). Do not normalize,
reinterpret, or replace with synonyms; keep them as-is whenever possible.
- Pay close attention to logical operators (AND, OR, IF); they control the analysis logic and outcomes.

Datasets:
- Allowed datasets: NHANES, AIREADI.

Inclusion/Exclusion rules:
- Include or exclude participants only according to the user's requested conditions.
- If criteria is redundant (both inclusion exclusion) just include it in either.
- Do not include/exclude based on missing values unless the user explicitly requests this for specific variables.
- For conditions that could be framed as either inclusion or exclusion, follow the user's framing.
- If the user wants to include all participants, set inclusion to None.

Analysis type:
- Choose one of: logistic regression, linear regression, cox regression, mediation analysis, group comparison, prevalence, stratified logistic
regression, weighted logistic regression, weighted linear regression, weighted cox regression, weighted group comparison, weighted prevalence,
weighted stratified logistic regression.
- If a weight type is specified, denote it in parentheses, e.g., "weighted logistic regression (exam)" or "weighted logistic regression
(questionnaire)". If no weight is specified, do not use a weighted analysis; the model will automatically select the most restrictive compatible
weights based on the variables used.
- If the analysis type is not specified, choose the most suitable one
- Variable requirements by analysis (list items in this exact order):
  - Logistic regression: covariates (if provided); predictor; outcome (binary mapped to 1 and 0).
  - Cox regression: covariates (if provided); predictor; outcome consisting of (1) time-to-event and (2) event indicator (binary, in this
order).
  - Linear regression: covariates (if provided); predictor; outcome.
  - Prevalence: grouping variables (if comparing groups); outcome (binary mapped to 1 and 0).
  - Mediation analysis: covariates (if provided), predictor; mediator; outcome.
  - Group comparison: variables being compared; outcome (the variable used to define groups).

Period of interest:
- Use the format YYYY-YYYY, e.g., 2014-2015.

This is an example of the input and expected output:

input will be:
{examples.question_free_text}

Output should be text with the following structure:
{examples.question}

Response to user: Output the final result wrapped in triple single quotes ('''   ''') with no extra commentary.
```

Objective: Convert the fully structured study design into a JSON object

```
Your goal is to create an organized dictionary for a study design based on the structured study design specification.

Rules for parsing input:
- Include full derivation details (e.g., categorization, binning, formula, IF/ELSE) without omitting any steps.
- For each derived variable, trace dependencies recursively until you reach the most basic variables, and list only these fundamental variables as
keywords.
- Include units in the keyword if present.
- For phrases that has conditions like missing value or more/less than certain value, organize the logic separately in keyword and condition so that
correct keyword can be later looked up.
- For analysis type, use the exact wording from thse input and don't omit anything.
- Make all keyword lowercase and no special symbols for consistency.
- For mapping derivations, list only the mapped labels, not the source-target pairs. Source values will be inferred later from examples.
- For mapping derivations, if any of the mapped labels is specified as reference keep the word "reference" in the final name for clarity.
- For categorical and binary variables in covariates, name them explicitly as group names in strings.

This is an example of the input and expected output:

input will be:
{examples.question}

Output should be a JSON object with the following structure:
{examples.parsed_question}

Response to user: Output the final result wrapped in triple single quotes ('''   ''') with no extra commentary
```

Supplementary Fig.1 | Prompt used in the Planner module. The LLM operates in two stages: first converting user free-text input into structured study design specifications and then transforming the structured output into a JSON object. A one-shot example is provided for each stage. The same prompt was used across all experiments in this study.

medRxiv preprint doi: https://doi.org/10.64898/2026.02.10.26346008; this version posted February 12, 2026. The copyright holder for this preprint (which was not certified by peer review) is the author/funder, who has granted medRxiv a license to display the preprint in perpetuity.

It is made available under a CC-BY 4.0 International license .

Variable Mapper

Objective: Among the given schema-based variable candidates, choose the variable whose context and description best align with the keyword from the user's input.

```
 Your goal is to select the most relevant candidate from the provided candidates that best aligns with the given keyword from the question.
 - If any candidate matches the keyword exactly from the user question, it is the correct candidate so select it.
 - If not, follow the instruction below to make a correct choice:
 - If more information like table name is available, use that to get more context and help you pick the most accurate candidate. For example, age
related information should be in demographic or patient related table, and serum levels should be in the lab or measurement table.
 - Make sure to be precise about any medical condition names. For example, pre disease X,  disease X, risk of disease X are different conditions pick
the correct one.
 - If there are multiple same name candidates, pick the most relevant one to the user's question based on the description.
 - If a keyword is related to a missing value or a comparison (e.g., less than or more than some value), pick the variable that is related to the
keyword. We can check for missing values or perform comparisons on that variable later. For example, for "Missing or Unknown vitamin C" or "vitamin C
level more than 10%", "vitamin C" is the correct choice.
 - Pick the one that most accurately describes what the original question is looking for.

 {example}

 Use only the provided options and do not introduce new ones.

 Response to user: The full formatted final output dictionary wrapped in triple single quotes (''' ... ''') without any explanation or commentary.
```

Supplementary Fig.2 | Prompt used in the Variable Mapper module. The LLM selects variables from schema-based candidates by matching the input keyword and original user text. A one-shot example is included. The same prompt was applied across all experiments in this study.

medRxiv preprint doi: https://doi.org/10.64898/2026.02.10.26346008; this version posted February 12, 2026. The copyright holder for this preprint (which was not certified by peer review) is the author/funder, who has granted medRxiv a license to display the preprint in perpetuity.

It is made available under a CC-BY 4.0 International license .

Data Engine

Objective: Generate a SQL query that uses inclusion and exclusion criteria to build the cohort, derives the requested variables in order, and outputs a final result table.

```
Your goal is to generate SQL code.
Follow these steps to answer the researcher's question, which is the user input.
You will generate a SQL query based on the user's question and the provided schema dictionary, which includes database tables, column names, and
example values.
Use PostgreSQL syntax.

SQL Writing Guidelines:

- Use "{patientid}" as the unique patient identifier.
- Always double-quote column names (e.g., "have_fever") to prevent errors.
- Use only valid column names from the schema.
- Use temporary tables to build the logic step-by-step.
- Add a clear comment above each temp table to describe its purpose.
- When categories are assigned through exact example matching, utilize allowed example strings explicitly and verify matches using strict string
equality.

Step-by-Step SQL Construction:

Step 1: Inclusion
- Create a temp table called temp_inclusion using rows from final_master_table that meet the inclusion criteria.
- If no inclusion criteria are provided, include all patients from final_master_table.
- For each inclusion rule, apply it in sequence using temp tables.

Step 2: Serial Exclusions
- If no exclusion conditions are provided in the user question, skip this step.
- For each exclusion rule, apply it in sequence using temp tables.
- Each exclusion must filter the output of the previous step.
- Do not subtract exclusion tables; instead, build one step upon the next.
- Start from temp_inclusion and apply each exclusion using a direct JOIN to final_master_table plus a WHERE clause.
- Do not automatically exclude records where the values are NULLS or UNKNOWN unless specified by the prompt.
- By default, if the exclusion criterion for variable ii is "valueX", we exclude only rows with ii = "valueX" and keep nulls. If the prompt explicit-
ly says to exclude missing ii and values "valueX", we exclude both.

Step 3: Final Cohort
- The final temp table in the exclusion sequence becomes temp_cohort.
- This cohort is used for all further computations.

Step 4: Variables
- Process variables in the order it was presented in the schema summary
- For each variable:
  - Join to temp_cohort.
  - Use intermediate temp tables if transformations, binning, calculations or conditional logic is required.
  - If binning (e.g., tertiles or quartiles) or formulas are needed, first create a temp table for raw values, then compute cohort-based cutoffs,
then assign group labels in a final predictor table.
  - For example-based categorization: do not use ILIKE/patterns/regex. Enumerate all allowed example strings and match exactly.

Step 5: Final Table
- Assemble temp_final_table by joining all temp tables on "{patientid}".
- The final table's variable's column order will be the following: {patientid}, {ordered_variables}

Step 6: Final Output
- End with: SELECT * FROM temp_final_table;

Example Reference

Schema:
{example_schema}

Example SQL:
{examples.sql_example(patientid)}

Your Schema Dictionary:
{schema_summary}

Response to user: Please output the final SQL query wrapped in triple single quotes ('''sql''') with no additional commentary.
```

Supplementary Fig.3 | Prompt used in the Data Engine module. The LLM follows a sequence of steps to generate and format SQL queries to ensure consistency and facilitate review. A one shot example is included. The same prompt was applied across all experiments in this study.

medRxiv preprint doi: https://doi.org/10.64898/2026.02.10.26346008; this version posted February 12, 2026. The copyright holder for this preprint (which was not certified by peer review) is the author/funder, who has granted medRxiv a license to display the preprint in perpetuity.

It is made available under a CC-BY 4.0 International license .

Dataset	Analysis	LATCH Input Query Example
NHANES	Prevalence	We will get a weighted prevalence of people with diabetes. We will use NHANES data from 1999-2008. Exclude people whose age at screening is less than 20, missing any of the following visual acuity values: presenting right visual acuity, presenting left visual acuity, post-refraction right visual acuity, post-refraction left visual acuity. We will also exclude people who are missing both of the following diabetes related information: glycohemoglobin (HbA1c) (%) and self-report of a doctor's diabetes diagnosis The main predictor will be a three-level categorical variable, 'vision group', defined using the better vision eye among right and left eye: "Group 1" No VI (Reference Group): Presenting visual acuity is 20/40 or better. "Group 2" Correctable VI (CVI): Presenting acuity is worse than 20/40, but post-refraction acuity is 20/40 or better. "Group 3"Nonrefractive VI (NVI): Presenting acuity is worse than 20/40, and post refraction acuity is still worse than 20/40. Outcome is having diabetes defined as meeting one of the criteria, having glycohemoglobin (HbA1c) (%) level of 6.5 or higher, who said yes to self-report of a doctor's diabetes diagnosis, categorized as binary 1 (having diabetes) and otherwise 0.
AIREADI	Logistic Regression	We plan to conduct a logistic regression analysis to examine the relationship between serum albumin (g/dL) and diabetic retinopathy using NHANES data from the years 2011 to 2020. The study will include all individuals whose age in years at screening was 30 years or older who have complete data on both serum albumin (g/dL) and diabetes affected eyes/had retinopathy. Participants will be excluded if their age in years at screening is less than 30 years old, or age when first told the patient had diabetes was before age 30, or had uncertain diabetes status. Uncertainty is based on their answer to the question "Has a doctor ever told you that you have diabetes?" and if they answer as "borderline" and either have a missing glycohemoglobin (HbA1c) value or an glycohemoglobin (HbA1c) below 6.5%. We will also exclude anyone with missing data on serum albumin or diabetes affected eyes/had retinopathy. The main predictor in the analysis will be serum albumin (g/dL), categorized into quartiles (%) Q1: <25, Q2: ≥25 and <50, Q3: ≥50 and <75, Q4: ≥75. The outcome variable will be the presence or absence of diabetic retinopathy, based on responses to the question "diabetes affected eyes had retinopathy?", coded as 1 for "Yes" and 0 for "No". We will adjust the analysis for potential confounding factors including gender, age in years at screening, and race/Hispanic origin, which will be categorized as White, Black, Mexican American, or Other.
AIREADI	Linear Regression	We will be performing a linear regression using AIREADI data from 2023-2025. We will exclude participants with missing study group, missing values in the following variables: mesopic log contrast sensitivity od, mesopic log contrast sensitivity os, glaucoma diagnosis, cataract diagnosis, diabetic retinopathy diagnosis, AMD diagnosis, retinal vascular occlusion diagnosis. The main predictor will be a study group and covariates are age, diagnosed with glaucoma, diagnosed with cataract, diagnosed with diabetic retinopathy, diagnosed with AMD, retinal vascular occlusion stroke in the eye. The outcome will be mesopic contrast sensitivity defined as higher one among mesopic od log contrast sensitivity or mesopic os log contrast sensitivity.
NHANES	Cox Regression	This study aims to investigate the relationship between serum uric acid levels (mg/dl) and all-cause mortality. We will be using NHANES data between 1999 and 2018, the analysis will employ a weighted Cox proportional hazards regression model and exam weights. The study cohort will be composed of participants age in years at screening 20 years and older who meet the criteria for diabetes when an individual is considered to have diabetes if they reported doctor told them they have diabetes, had a glycohemoglobin A1c (HbA1c) level >= 6.5%, or a fasting plasma glucose level (mg/dl) is 126 mg/dL or greater. Individuals will be excluded from the analysis if their pregnancy exam was positive. Participants with missing laboratory values for serum uric acid (mg/dl), serum creatinine (mg/dl), or glycohemoglobin (HbA1c) (%), will be excluded. Anyone who reported ever being told they had cancer or a malignancy will be excluded, as well as those missing mortality data or months followup examination, will be excluded. The primary predictor for this analysis is the serum uric acid level (mg/dl), which will be categorized into five groups based on its distribution in the study population, using quintiles as cut-offs QN1: <20%, QN2: >=20 and <40%, QN3: >=40 and <60%, QN4: >=60 and <80%, QN5: >=80%,The main outcome is all-cause mortality, categorized as 1 or 0, with months followup examination serving as the time-to-event variable. The analysis will control factors, including age in years at screening, gender, and race/Hispanic origin, which is categorized as Non-Hispanic White, Non-Hispanic Black, Mexican American or Hispanic, and Other.

Supplementary Table 1 | Examples of text inputs to the LATCH framework.

medRxiv preprint doi: https://doi.org/10.64898/2026.02.10.26346008; this version posted February 12, 2026. The copyright holder for this preprint (which was not certified by peer review) is the author/funder, who has granted medRxiv a license to display the preprint in perpetuity.

It is made available under a CC-BY 4.0 International license .

Analysis	Figure	Ref	Analysis Type	Reported Values	Reproduction Assessment Bases	Concordance
Monocyte to Lymphocyte Ratio → Diabetic Retinopathy	Fig. 2a	25	Logistic Regression	OR, 95% CI, p value	Effect Direction, CI Overlap, P value significance,	Yes
Albumin → Diabetic Retinopathy		26				Yes
HDL Cholesterol → Diabetic Retinopathy		27				Partial
Lactate Dehydrogenase → Diabetic Retinopathy		28				Yes
Hemoglobin → Diabetic Retinopathy		29				Yes
UACR/SACR Ratio → Diabetic Retinopathy		30				Yes
Systematic Inflammation Index → Diabetic Kidney Disease		31				Yes
Neutrophile to Lymphocyte Ratio → Diabetic Kidney Disease		33				Partial
Weight Adjusted Waist Index → Diabetic Kidney Disease		34				Yes
Folate → Diabetic Kidney Disease		35				Yes
Food Insecurity → Depression in Diabetes		38				Yes
Neutrophile to Lymphocyte Ratio → Depression in Diabetes		39				Yes
Depression Severity → Diabetes		40				Yes
Uric Acid → Mortality in Diabetes	Fig. 2b	41	Cox Regression	HR, 95% CI, p value	Effect Direction, CI Overlap, P value significance,	Yes
Stress Hyperglycemia Ratio → Mortality in Diabetes		42				Partial
Red Blood Cell Width → Mortality in Diabetes		43				Yes
Kidney Disease in Diabetes	Fig. 2c	32	Prevalence	%	Absolute Difference within 1%	Yes
Depression in Diabetes		36				Yes
No Retinopathy vs Diabetic Retinopathy	Fig. 2d	24	Group Comparison	Mean, Standard Deviation	Direction of between-group differences, CI Overlap	Yes
Glycohemoglobin → Cognitive Test	Fig. 2e	37	Linear Regression	B, 95% CI, p value	Effect Direction, CI Overlap, P value significance,	Partial

Supplementary Table 2 | Summary of the 20 studies included in the LATCH reproduction analysis. Analysis refers to the analysis name reported in Fig. 2. The Ref column indicates the corresponding publication number listed in the references. The Figure column corresponds to the subfigures in Fig. 2 representing each reproduced analysis. Reported Values are the results originally published in each study, whereas Replication Assessment Bases are the metrics used to evaluate the extent of reproduction. Concordance indicates the overall reproduction outcome: Yes if all reproduction assessment bases were fulfilled; Partial if one or more criteria showed discrepancies; and No if none of the criteria were met.

medRxiv preprint doi: https://doi.org/10.64898/2026.02.10.26346008; this version posted February 12, 2026. The copyright holder for this preprint (which was not certified by peer review) is the author/funder, who has granted medRxiv a license to display the preprint in perpetuity.

It is made available under a CC-BY 4.0 International license .

LATCH Module	Safeguard Activation Condition	Safeguard Message Example
Planner	User input requires clarification due to missing, contradictory or ambiguous information	Review Required. This analysis plan is mostly well-defined, but there are a few points that need clarification to ensure the analysis can be conducted accurately: Exclusion Criteria Ambiguity: The exclusion criterion "Participants will be excluded if they are really sick and missing a lot of information" is too vague. Please specify how "really sick" will be defined (e.g., based on specific medical conditions, number of missing variables, or a particular health status indicator) and what constitutes "a lot of information" (e.g., a threshold for missing data points).
		Review Required. The requested "machine learning analysis" is not one of the supported analysis types (logistic regression, linear regression, cox regression, group comparison, prevalence, or mediation analysis). However, based on your description of a binary outcome (presence or absence of diabetic retinopathy), a predictor (serum albumin), and several covariates, a **logistic regression** would be the most appropriate statistical analysis to examine the relationship you've described. Please confirm if a **logistic regression** would be an acceptable analysis type for your study.
Variable Mapper	Inconsistencies are detected in the variables matched to user-input keywords across cycles	Multiple variables candidates detected for 'A1C ≥ 6.5%' across cycles: ['glycohemoglobin', 'what_was_your_last_a1c_level'] Searching for a candidate available in all cycles...Found a candidate that is available from all cycles: 'glycohemoglobin'(From: Diabetes - diq_g) If you prefer to use other candidates, consider adjusting your time period and replace A1C ≥ 6.5% with other candidate. Rebuilding results for 'A1C ≥ 6.5%' using 'glycohemoglobin'.
		Multiple variables candidates detected for 'renal albumin loss' across cycles: ['albumin_urine_ug_ml', 'albumin_creatinine_ratio_mg_g'] Searching for a candidate available in all cycles...Skipping candidate 'albumin_creatinine_ratio_mg_g' (From: Albumin & Creatinine - Urine - alb_cr_f). Missing from cycles: [(2007, 2008), Found a candidate that is available from all cycles: 'albumin_urine_ug_ml' (From: Albumin & Creatinine - Urine - alb_cr_f) If you prefer to use other candidates, consider adjusting your time period and replace renal albumin loss with other candidate. Rebuilding results for 'renal albumin loss' using 'albumin_urine_ug_ml'.
Data Engine	SQL did not run due to execution error (iterative correction up to three times)	The error "syntax error at or near "do"" occurs because `do` is a reserved keyword in PostgreSQL.In the `CREATE TEMP TABLE temp_final_table` statement, the alias `do` is used for `temp_depression_outcome`. To fix this, either quote the alias as `"do"` or change the alias to a non-reserved word (e.g., `tdo`). Replace `do."depression_outcome"` with `"do"."depression_outcome" ...{Fixed SQL Query}
		The error `function percentile_cont(numeric, text) does not exist` indicates that the `PERCENTILE_CONT` function is being called with a `text` type argument in its `ORDER BY` clause, but it expects a `numeric` type. ...{Fixed SQL Query}

Supplementary Table 3 | Examples of messages generated by safeguards implemented in the Planner, Variable Mapper, and Data Engine modules.
